# The Resonance Frequency Shift, Pattern Formation, and Dynamical Network Reorganization via Sub-Threshold Input

**DOI:** 10.1371/journal.pone.0018983

**Published:** 2011-04-19

**Authors:** Troy Lau, Michal Zochowski

**Affiliations:** 1 Department of Physics, University of Michigan, Ann Arbor, Michigan, United States of America; 2 Biophysics Program, University of Michigan, Ann Arbor, Michigan, United States of America; Newcastle University, United Kingdom

## Abstract

We describe a novel mechanism that mediates the rapid and selective pattern formation of neuronal network activity in response to changing correlations of sub-threshold level input. The mechanism is based on the classical resonance and experimentally observed phenomena that the resonance frequency of a neuron shifts as a function of membrane depolarization. As the neurons receive varying sub-threshold input, their natural frequency is shifted in and out of its resonance range. In response, the neuron fires a sequence of action potentials, corresponding to the specific values of signal currents, in a highly organized manner. We show that this mechanism provides for the selective activation and phase locking of the cells in the network, underlying input-correlated spatio-temporal pattern formation, and could be the basis for reliable spike-timing dependent plasticity. We compare the selectivity and efficiency of this pattern formation to a supra-threshold network activation and a non-resonating network/neuron model to demonstrate that the resonance mechanism is the most effective. Finally we show that this process might be the basis of the phase precession phenomenon observed during firing of hippocampal place cells, and that it may underlie the active switching of neuronal networks to locking at various frequencies.

## Introduction

Sub-threshold oscillations are ubiquitous throughout the brain and span wide range frequencies. While the sources of these oscillations are not well understood, they are known to originate from various brain regions, and thus have different cognitive function depending on their spectral properties [1]. Some of these oscillations maybe generated by intrinsic neural oscillators, others are thought to originate from network interactions. For example, theta rhythms (6–10 Hz) originate in hippocampus and have been shown to correspond to the ‘active learning’ state [2], [3]. Theta rhythms have been implicated in learning and the encoding of memories [4]–[6]. These oscillations, along with synaptic modification via spike-timing-dependent plasticity (STDP) provide the necessary basis for the formation and changes of memory traces in neuronal networks of the brain [6]–[8]. At the same time, cortico-cortical and thalamocortical networks are implicated in generation of alpha rhythms (8–12 Hz) [9], while beta rhythms are mostly generated in motor cortex. Gamma rhythms (20–80 Hz) are widely distributed over the cortex and are thought to be mediated by fast-spiking inhibitory interneurons. Their function is still not well understood but one possible implication is in controlling sensory responses [10].

At the same time it has been demonstrated that certain types of neurons have the ability to resonate [11], [12] and fire in response to a specific sub-threshold oscillatory current. Furthermore, it has also been recently shown that this natural frequency can shift in response to changes in the neuron's membrane potential [13], [14]. Here we propose a novel mechanism linking these three experimentally observed phenomena in which a neuronal network may utilize intrinsic oscillatory patterning, together with cell's ability to resonate and dynamically shift its resonant frequency, as a means to encode patterns based on the characteristics of a **sub-threshold** signal current. We show that changing the magnitude of the sub-threshold input can shift the cells' natural frequency into, and out of, the sub-threshold oscillatory current's range. This causes the neuron to resonate and phase lock to the period of the oscillation when the signal current is within a certain range. We use a network of resonate-and-fire (RAF) [15] neurons to demonstrate that this mechanism generates a highly selective spatio-temporal firing pattern. We compare the response properties of this network to a supra-threshold stimulated RAF network and to a network of supra-threshold stimulated integrate and fire neurons (IAF), all receiving sub-threshold oscillatory currents. We show that the RAF frequency adaptation mechanism is far superior at resolving temporal correlations/differences than the other models. This property, in conjunction with spike timing dependent plasticity (STDP), can be utilized to store temporal correlations between different input. Finally, we use this natural frequency shift mechanism to explain two experimentally observed phenomena in the hippocampus: the phase precession [16], [17] along theta oscillation observed in the firing of hippocampal place cells as animal traverses its place field, and the dynamic changes in phase locking observed between the medial prefrontal cortex and the ventral or dorsal hippocampus during fear or a working memory task respectively [18], [19].

## Methods

### Resonate and fire neuron

To investigate the performance of proposed resonance adaptation mechanism we used a network of 200 randomly coupled, excitatory, resonate-and-fire neurons [15], [20]. The neurons are described by a set of two ordinary differential equations representing the internal current (*x*) and voltage (*y*) of the cell.
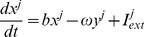
(1)

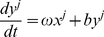
(2)


Where, for neuron 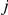, 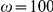 modifies the natural oscillation frequency, 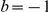 defines the attraction of the voltage to it's resting potential, and 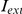 is the external current defined as
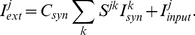
(3)


Here, the first term is the synaptic current received from other firing neurons; 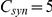 is the synaptic coupling strength, 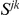 is the synaptic connectivity matrix. The synaptic coupling is defined as
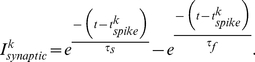
(4)


Here, 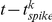 is the time since the pre-synaptic neuron firing, 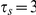 and 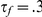. The variables 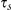 and 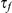 are chosen such that the post synaptic potential has a pulse shape and lasts approximately 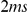. The second term, 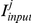, denotes external current.

After each neuron fires at 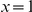, x is reset to 0 and held there for 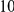
ms – the duration of the refractory period.

Based on experimental results [13], [14] the resonant frequency shift is set to be a linear function of the total external current received by the given cell,
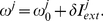
(5)


**Figure 1:**
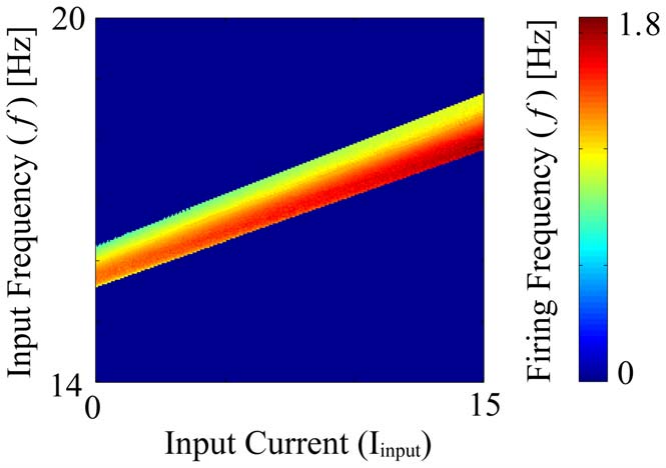
Firing frequency response of a single neuron to varying strengths of the signal current and frequencies of the oscillatory current. The oscillating current amplitude is fixed at 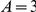 and 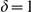. The color scale denotes the firing frequency of a neuron.

Here 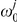 is the oscillation frequency in the absence of any external currents, and 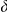 is a scaling factor. Figure 1 demonstrates the resonance response of the neuron for different signal currents and sub-threshold current frequencies. Experimental studies have demonstrated both positively and negatively sloped responses to neuron depolarizations 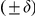 [13], [14]. We have chosen 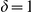, however both responses will produce similar results.

The input current consists of two components and is defined as
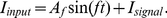
(6)


The first component is a sub-threshold oscillatory current of amplitude 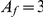. For 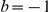 and 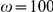 the resonance frequency is between 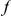 = 15–19 Hz (see above figure) thus we used 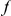 = 17 Hz as our primary input frequency. This frequency can be easily adjusted without changes to the described behavior. The second component was a sub-threshold (except when compared with **supra-threshold** resonate and fire network) current input to the network (e.g. a sensory input). The specific properties of the input signal are defined in detail in the next section, however note that the maximum magnitude of 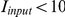, whereas the current threshold needed for the cell to fire, defined by Equation 1, is around 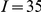. Thus, for the sub-threshold resonate and fire network, the total input current is well in sub-threshold regime at all times.

### Integrate and fire neuron

To compare the results from the RAF model to another easily tractable model we used the leaky integrate-and-fire neuron model:
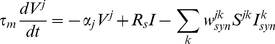
(7)


Here, 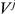 is the membrane potential of the 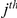 neuron, 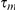 = 0.5 ms is the time constant; 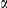 is a leakage coefficient which is different for every cell, 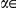 [1∶1.3]; 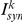 is the synaptic current generated at the time of the spike, 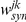 defines the chemical synapse coupling strength; 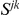 is the synaptic connectivity (adjacency) matrix; 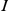 is a uniform external current which keeps the neurons readily excitable, 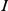 = 0.5; 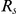 is the neuron resistance 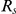 = 1.

The synaptic current is activated after the pre-synaptic neuron reaches a threshold 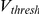 = 1 and fires an action potential. The pre-synaptic neuron is then returned to 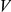 = 0 and remains there for a refractory period 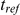 = 10 ms. The synaptic current is of the form
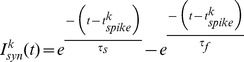
(8)where 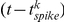 is the time since the last firing of the presynaptic neuron, 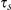 = 3 ms is the slow time constant, and 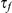 = 0.3 ms is the fast time constant. The variables 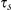 and 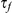 are chosen such that the post-synaptic potential lasts approximately 2 ms.

### Measuring temporal pattern properties: mean phase coherence

We used the mean phase coherence (MPC) to measure the amount of phase locking between cells [15], [21]. The MPC ranges between 0 (no phase locking) and 1 (maximal phase locking). The MPC is calculated pair-wise between neurons 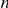 and 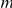:
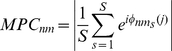
(9)


Here 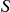 is the total number of spikes of cell m and 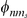 is the phase between cell 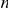 and 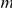 for interval 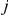 containing 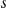. This phase is defined as:
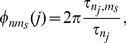
(10)where
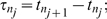
(11)is the inter-spike-interval 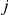 for neuron 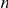 containing spike 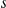 of the 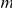-th cell and
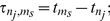
(12)is the time difference between the initial firing of neuron 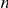, on interval 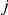, and the firing 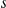, of neuron 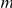, with the condition,
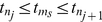
(13)


Finally, we take the average of all MPC pairs across all neurons,
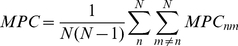
(14)where 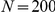 are the total number of neurons.

### Signal Phase Coherence

We also measure phase coherence of the neurons with respect to the oscillatory drive. Here the phase of the oscillatory signal at which given cell fired was obtained directly. The signal phase coherence was calculated in a similar fashion to the MPC.

### Mean minimal interneuron interspike interval

To further quantify the temporal spiking pattern between the neurons we calculated mean minimal interneuron interspike interval (mISI). Namely we calculated the ISI length for the nearest firing times between every neuron:
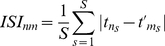
(15)where, 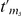 is the nearest firing of cell 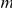 to 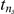.

Then
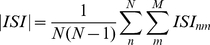
(16)is the mean inter-neuron ISI.

## Results

**Figure 2:**
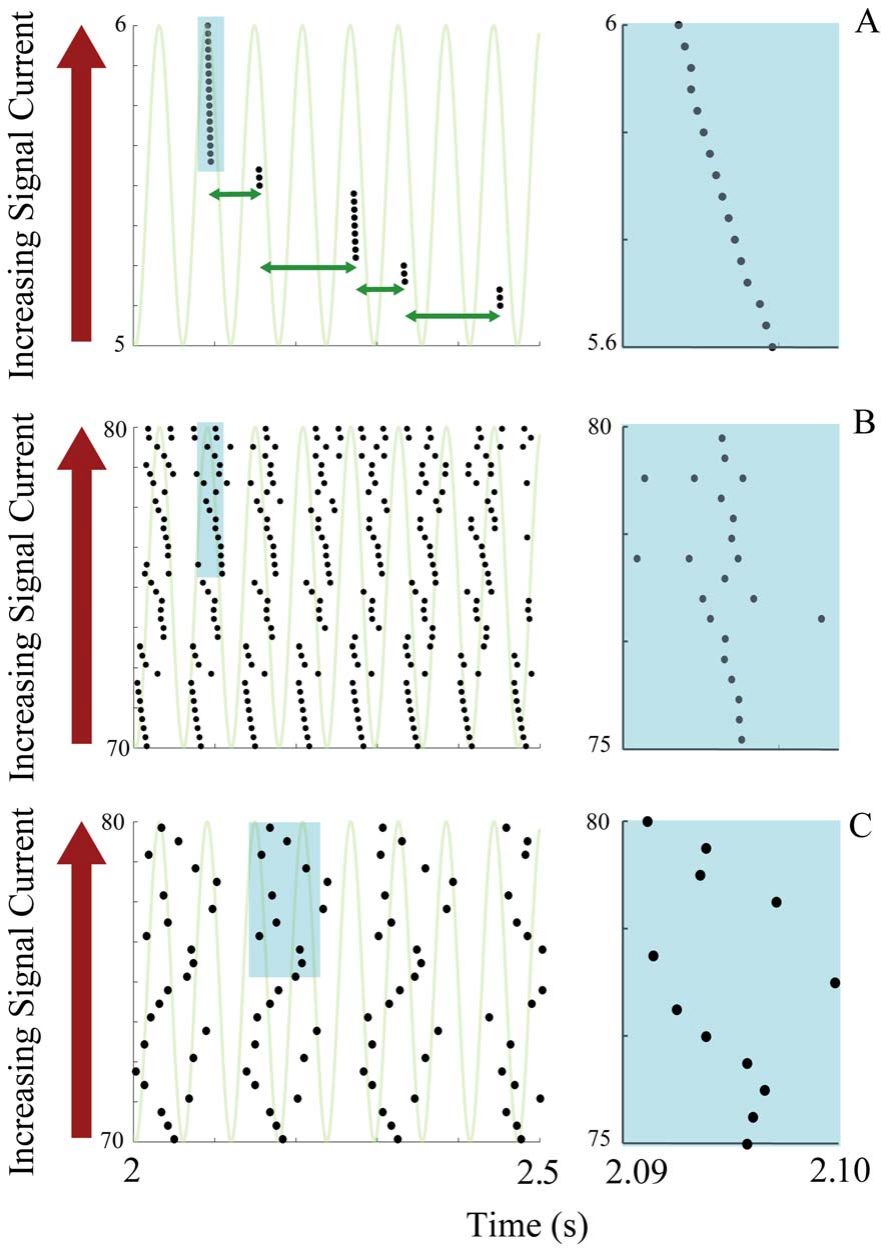
Raster plot of neuronal responses of neurons to a range of input signal currents compared across different models. The neuron id/current (y-axis) vs. time (x-axis) is plotted where stars represent the neuronal firing times. The neurons are uncoupled here to best demonstrate their firing pattern response. The resonant RAF model is plotted in A, the supra-threshold RAF model in B, and the supra-threshold IAF model in C. The neurons were ordered so that the signal current to a neuron increases with its id. The oscillatory current is overlayed with the spike times to demonstrate the phase-locking of the neurons to the oscillation. A) Neurons receiving similar currents fire in a temporal order dependent directly on the value of the input current they receive (shaded regions). Neurons receiving significantly different currents fire on different oscillatory cycles, remaining phase locked to the oscillation, but not to each other. B and C) Neurons fire erratically, not phase locked to the resonant oscillations, or in any particular order corresponding to the signal current.

We compared the performance of the RAF resonant frequency adaptive network with two other network realizations: an identical RAF network driven by a supra-threshold signal current, and a non-resonating IAF [22] network driven by a supra-threshold signal current. All networks received a fixed sub-threshold oscillatory current with a frequency of 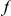 = 17 Hz and an amplitude of 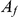 = 3. The examples of the raster plots and the relation of spike timing to the underlying oscillation for all three networks are shown on Figure 2.

### Comparison of neuronal and signal phase locking properties

**Figure 3:**
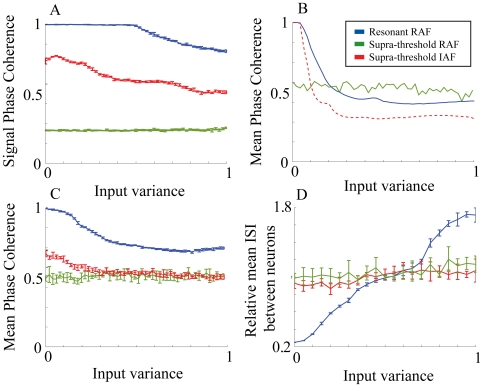
Response of neurons to a range of input signal currents. The RAF resonance adaptation mechanism with sub-threshold input is denoted by blue, RAF with supra-threshold input by red, and IAF with supra-threshold by green. a) The phase coherence of the network firing times with the oscillatory current v.s increasing ranges of signal currents. b) The MPC response of an uncoupled network to increasing ranges of signal currents. c) The MPC response of a coupled network to increasing ranges of signal currents. d) The mean inter-spike interval (ISI) calculated between neurons vs. increasing ranges of signal currents for a coupled network. Errors are calculated over five simulations.

First we examined the response of the networks to a range of different input currents. We do this by investigating the degree of selectivity and locking of network activity as a function of the variance of the input (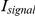). Here, the magnitude of the input current was drawn from a random Gaussian distribution to vary the signal currents into each neuron. To keep the relative variance range (between the sub and supra-threshold signal currents) the same, the subthreshold amplitude of the signal current, (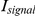), had a mean 6 and maximal variance of 3, while the supra-threshold currents amplitude had a mean of 80 with the maximal variance of 40. These maximal variances correspond to 1 on the x-axes of plots on Figure 3. For each simulation, the specific value of the signal current was kept constant over time. We computed the mean phase coherence, phase locking of activity to the oscillatory current, and the mean inter-neuron ISI for the three types of networks.

Figure 3A depicts the phase locking of neuronal activity to the network oscillatory drive as a function of the input variance. One can observe that the phase locking for the frequency adaptation network is nearly perfect for most of the range, tailing off when the subthreshold 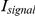 variance approaches half of its maximal value, or 1.5. This indicates that the neurons are locked to specific phases on the oscillatory current. This is due to the fact that the active neurons (i.e those that receive appropriate current shifting their natural frequency towards the frequency of the oscillatory drive), being effectively oscillators, phase synchronize with the oscillatory current [15], [23], [24]. Other neurons remain quiescent as they do not enter resonant firing - their natural frequency is significantly different from that of the oscillatory drive while the total input signal they receive is sub-threshold. Figure 2A demonstrates this behavior and shows that the firing times are locked near the peaks of the oscillation, overlayed in gray. This effect is significantly diminished for the RAF network with supra-threshold input (B), and almost completely absent in the IAF network (C). It occurs because the neurons' firings are effectively driven by the supra-threshold inputs with the cell firing frequency determined by the amplitude of this input.

Figures 3B and 3C depict the MPC changes of uncoupled neurons and coupled networks, respectively. The MPC is an indicator of the stability of the phase relationships between the neurons themselves. This, in turn, determines stability and selectivity of the generated network activity pattern. The MPC for the uncoupled adaptive RAF network is shown in blue in Figure 3B. Here the MPC is high for low input variance but declines quickly as the input variance is increased. This is in contrast to the signal coherence in 3A because, even though the neurons are locked to the phase of the individual oscillatory cycle, they fire at different cycles, depending on the signal current magnitude. Figure 2 demonstrates this effect in the shaded region. Here we see the timing of neurons' firing, on a specific oscillatory cycle, as a function of input signal, where neurons with similar 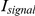 fire near synchronously with a consistent phase relationship to the oscillatory signal. Neurons with significantly different input currents however, fire on different oscillations of resonant current (shown by different green arrow lengths). When the neurons are coupled (Figure 3C), the excitatory connections mediate increased neuronal interactions and firing at the same oscillatory cycle leading to a higher MPC. By comparison, for both the coupled and uncoupled case, the supra-threshold RAF and IAF networks have lower MPC. For the supra-threshold RAF neurons the MPC remains high for a narrow range of signal currents because of the phase locking of the cells receiving similar input, however for larger values of the variance the differences in the 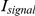 lead to significantly different firing frequencies, no locking to the oscillatory current and thus abolishing of phase locking (Figure 2). For the IAF model the MPC remains low over all input variance range, because the non-oscillating neurons lack and frequency response properties.

Finally, Figure 3D depicts the modulation of the inter-neuron inter-spike intervals (ISI) as a function of variance of input currents. The mean ISI changes significantly for the resonance adaptation mechanism, while it remains constant for other two models. This indicates an increased signal current selectivity (in terms of spiking coincidence), as a function of input variance, for the adaptive resonance mechanism compared with supra-threshold input for both RAF and IAF models. This occurs because, for small values of variance, the active cells fire within narrow time windows. When the variance is increased the cells are still locked to the oscillation phase but are firing on different oscillatory cycles, rapidly increasing the mean ISI value. Again, we can observe this on Figure 2 where, as the current deviates further from the peak of 6, the pair-wise ISIs increase more and more. This effect is abolished for the other two network realizations as the supra-threshold inputs inhibit cells from phase locking and thus the specific variance of input has little effect on the ISI. As we will show below, this phenomenon has a large effect on the efficiency of the STDP driven synaptic modifications.

### Specific effects of resonant frequency shift vs. different sub-threshold currents

#### Enhanced STDP driven synaptic modifications and the spatio-temporal correlation of inputs

The results described above indicate that the resonance frequency shift provides a superior mechanism to translate differences in the input signal characteristics to distinct patterns of spatio-temporal neuronal activity. Next we investigate how well these neuronal activity patterns translate to STDP modified network connections. To do this we used a standard symmetric decaying exponential learning rule to model the effects of STDP on the network. Here the synapses may be strengthened (depressed) by a factor 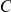 if the presynaptic neuron fires shortly before (after) the postsynaptic cell:
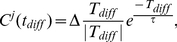
(17)where 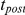 and 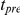 denote time before and after synaptic modification, respectively; 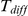 is the time difference between the presynaptic and postsynaptic neuron firing, 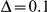 scales the STDP strength, and 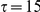
ms is the STDP time constant defining the relevant timescale for synaptic changes.

**Figure 4:**
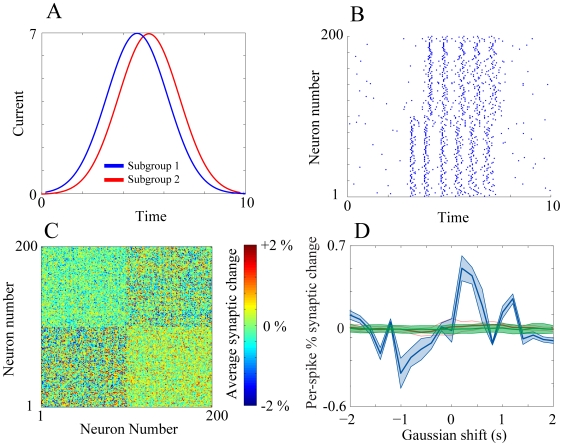
STDP network connectivity changes due to the correlation of Gaussian inputs. A) The Gaussian profile and temporal shift of the signal currents into each region, here 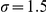
s, 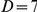 for sub-threshold input and 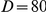 for supra-threshold input. B) Example of the activity raster plot of neuronal activity. C) The pattern of modified connectivity strengths between the neurons at the end of the simulation (10 s), averaged over 100 trials, for all possible connections. x-axis is from, y-axis it to. D) Comparison of mean, directional connectivity changes between the two network subgroups as a function of the input shift. The different colors are a comparison between the RAF adaptive resonance network, supra-threshold RAF network, and supra-threshold IAF network.

The network was divided into two sub-groups (id. 1–100, 101–200) each receiving a signal current with time-shifted Gaussian profile,
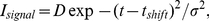
(18)where 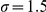
s, 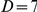 for sub-threshold input and 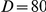 for supra-threshold input, Figure 4A. For the data depicted on Figure 4A–C the time shift between the two sub-groups was fixed at 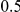
s. The two Gaussians represent distinct activation fields by which the two subpopulations respond to (e.g two nearby place fields that activate two subpopulations sequentially, as an animal runs through the maze). Figure 4B depicts the spike timing raster of the network, and Figure 4C shows the resultant connectivity matrix, obtained at the end of the simulation (t = 10 s), averaged over 100 trials. We see that, within each sub-region, where neurons receive identical inputs, strong increases or decreases occur in the synaptic strengths. These changes are symmetrical (the net changes average to zero), with the specific patterning of STDP changes governed by the initial random connectivity. More importantly however, one can observe a strong unidirectional strengthening (weakening) of connections from regions with the leading(following) Gaussian. This is due to the fact that the subset receiving to the leading Gaussian initially receive a slightly higher current, in the resonant range, compared to the subset receiving the lagging Gausssian. When these differences are small, this leads to robust phase shifts in firing within the same oscillatory cycle, between the two populations (Figure 2).

Finally, we investigated how the unidirectional coupling changed as a function of the temporal shift between the Gaussian signal currents. We did this for all three neuron/network models by computing the difference between the mean couplings of both regions. This will directly measure the extent to which the changes in the network topology reflect the correlation between the signal currents. Figure 4D depicts the normalized (per spike) changes in directional connectivity between the two neuronal sub-groups for the RAF frequency adaptation network with sub-threshold input (blue), supra-threshold input (red), and the IAF network with supra-threshold input (green). Clearly the sub-threshold input, together with the resonant frequency adaptation mechanism, provides the most supportive dynamical environment for the network reorganization. The changes are reflected in directional connectivity between the two regions, correlating the time dependence of the signal currents to the strengthening of connections.

## Discussion

Based on the results above, it is clear that the sub-threshold driven resonant neurons, coupled with a voltage dependent natural frequency shift, provide a very efficient dynamical mechanism for the formation of input driven spatio-temporal patterns of activity. This, integrated with STDP learning, provides an efficient mechanism that underlies the formation of a connectivity topology that maps the temporal and spatial characteristics of the input signal(s) - more so than supra-threshold input driven networks. This effectiveness arises from the enhanced phase and signal locking, due to the resonance frequency shift response, and the higher sensitivity in spike timing due to resonance induced firing. In short, the neurons' firing times are consistently mapped onto specific, current dependent, phases of the input oscillation, rather than just being modulated by a supra-threshold oscillating current.

### Input dependent phase precession

**Figure 5:**
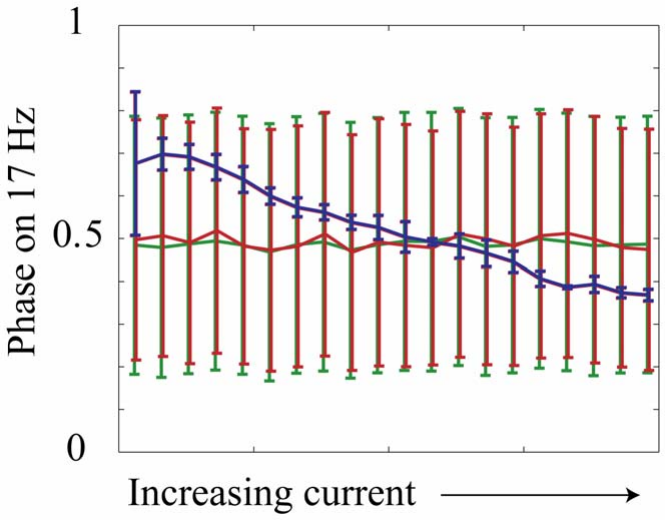
Phase precession as a function of input strength. The lines denote average phase of neuronal firing with respect to the oscillatory current (blue – sub-threshold resonance adaptation mechanism; green - supra-threshold input driven RAF; red – supra–threshold driven IAF).

This input-dependent phase locking and phase precession has been observed experimentally in nearly all parts of the brain involved in learning [6], [25], [26], and specifically during hippocampal place cell firing [16], [17] when the animal is traversing the place field associated with that cell. While it is relatively difficult to explain this phenomenon using supra-threshold network realizations, it is an intrinsic property of the sub-threshold resonance adaptation mechanism we described Figure 5.

### Dynamic modulation of information transfer between brain modalities

**Figure 6:**
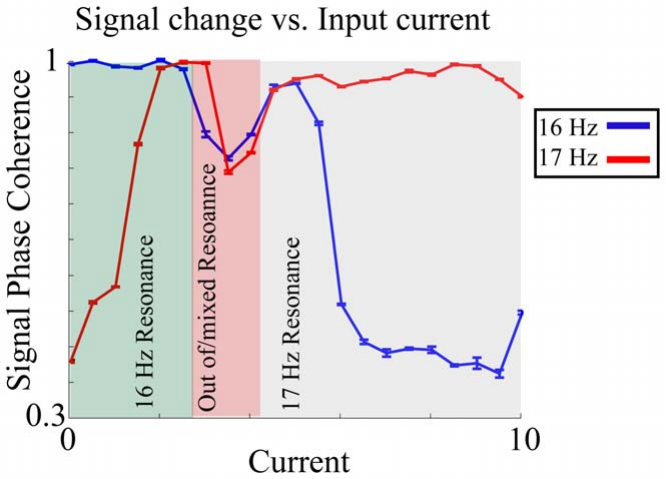
Dynamic changes in phase locking between two oscillatory inputs. The network is driven by two oscillatory inputs having different frequencies (16 Hz and 17 Hz). Signal coherence is measured with respect to both signals (red and blue lines). As the 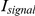 is increased the network switches locking from one to other signal.

Finally, the voltage dependent natural frequency shift may explain recently observed dynamic changes in information flow between different brain modalities. It has been shown that the medial prefrontal cortex synchronizes with the ventral hippocampus (vHPC) during anxiety [18] and with the dorsal hippocampus (dHPC) during working memory tasks [19], specifically in the theta (4–12 Hz) range in both cases. It is also known that the dHPC and vHPC have slightly different preferred frequencies of theta that route the flow of information in different states. Such a dynamic change in frequency preference between modalities is easily explained within our model. While it is not clear what, in this case, causes direct additional cellular depolarization creating the resonant frequency shift, it was shown that in 5HT1A KO mice (i.e. serotonin receptor knock-outs, a model for increased anxiety), that there was an increased theta power increases over wild type [18]. Since the knock-out of 5HT1A receptor has depolarizing effect it could provide the mechanism for the proposed resonant frequency shift leading to increased theta frequency. Figure 6 depicts such a transition for the frequency ranges we used earlier. Here the network receives two oscillatory inputs with slightly different frequencies. As the cells' membranes are progressively depolarized the network shifts from being locked to the lower frequency input to the higher frequency one, as reported by the signal coherence.

To the best of our knowledge, we are the first to demonstrate the use of oscillations and the sub-threshold frequency shift as a mechanism which provides brain networks with the enhanced ability to encode input patterns.
